# Clinical Lecture on Stone in the Bladder

**Published:** 1831-01

**Authors:** 


### STONE IN THE BLADDER.

*Clinical Lecture on Stone in the Bladder.* By Mr. Mayo.

Mr. Mayo observed that, during the last summer and autumn, he had operated, in the Middlesex Hospital, upon four patients afflicted with stone; and that he proposed to make their cases the groundwork of some observations upon particular points in the treatment of that malady.

The first case to which Mr. Mayo referred, has been already published in our Hospital Reports, with some remarks upon the method of operating, which were repeated in the lecture. In this instance the calculous matter formed round a broken catheter. Two circumstances there were, though neither very strongly marked in this case, which Mr. Mayo remarked upon as deserving consideration. The first was a difficulty met with in ascertaining the presence of the foreign body in the bladder; the second was a partial incontinence of urine, experienced by the patient for some time after he had recovered from the operation, showing itself first when he began to move about again.

With reference to the first point, it is extremely useful, according to Mr. Mayo, to sound a patient, not merely in two positions, and in two states of the bladder, but likewise with two instruments successively: one, the common sound, with its long bold curve, that is particularly adapted to sweep the fundus of the bladder; a second, that of Civiale, perfectly straight, with the exception of the last inch and half which is abruptly curved, and well fitted to explore with more precision the surface adjacent to the neck of the bladder.

With respect to the incontinence of urine, observed after the operation for the stone, this results partly from some remaining inflammation of the mucous membrane of the bladder, partly from the acquired habit of the detrusor urinæ to contract immediately that a given degree of distention has taken place. In the case adverted to, this symptom disappeared under the use, first, of decoction of uva ursi with liquor potassæ, and afterwards of pills containing carbonate of iron and Chian turpentine. There are mechanical methods of accustoming the bladder gradually to retain more and more urine: either a jugum on the penis, the incautious use of which however is liable to produce swelled testis, the urine being stopped in and irritating the urethra; or, which is better, a light truss making pressure upon the perineum, and keeping the confined urine within the bladder.

The second case was that of a man sixty years of age, who had laboured six years under the stone, and who, though long unwilling to submit to the operation, had at last been induced, by the severity of his sufferings, to solicit its performance. The stone was of a large size, and the symptoms were strongly marked;—the pain most intense just after the bladder is emptied, lasting for several minutes, and seeming to originate in the bladder, then to shoot along the urethra, and to be fixed, with intenser aggravation immediately within the frænum;—the urine, sometimes while flowing, suddenly stopped;—frequent micturition;—the urine clouded with mucus, and, at last, containing thick adhesive muco-purulent secretion, of a highly ammoniacal odour;—after exercise, blood passed with the urine, and all the symptoms aggravated; together with a distinct and painful sensation of the presence of the stone, on any bodily movement. To make the detail of symptoms complete, the patient commonly recollects that four or five months before those symptoms began, with which he is now afflicted, he was ill with pain across the loins, extending in the course of the ureter towards the bladder; and that, after three or four days' suffering of this description, the pain in the back subsided; upon which, he passed bloody water, and laboured under irritation of the bladder, with frequent and painful micturition, for three or four days more; then became, to all appearance, well.

But the case at present referred to had more serious interest. The stone was of large size, being six ounces two drachms in weight; and of an unlucky figure, not being oblong, but circular and unequally flattened, requiring, for its exit out of the bladder, an aperture three inches by one inch and a half. The operation occupied rather more than half an hour: to facilitate the extraction of the stone, Mr. Mayo divided the neck of the bladder on both sides. The patient bore the operation with perfect fortitude, but he survived it only twenty-four hours. Mr. Mayo remarked, that he would not operate again in a similar case, without having at hand an instrument sufficiently powerful to break a stone in fragments, if, after opening the bladder, the shape or size of the stone should appear to render the exertion of considerable force requisite in its extraction.

The next case adverted to was that of a patient, of the name of Wareham, forty-two years of age, who, two years ago, was cut by Mr. Wardrop. About eight months after the first operation, symptoms of the stone again showed themselves: there was great irritation in the bladder. The second operation was performed in September: a small flat stone was extracted. The patient did well.

The fourth patient was Thomas Hearne, ætat. twenty-one; the stone extremely small and its figure irregular, from previous lithotritic operations. The wound closed rapidly, and the urine flowed again entirely by the urethra on the eighth day.

The two last cases presented two points of considerable interest in common. In both some hemorrhage occurred after the operation, and in both lithotrity had been attempted.

With reference to the occurrence of hemorrhage: in Wareham's case, about ten ounces of blood were lost in the operation; an hour afterwards, blood began to flow again from the bottom of the wound, and from eight to ten ounces more were lost. The bleeding was arrested by inserting through the wound a silver catheter, with a plug of lint rolled round it. This was removed the following day. Eleven days after, when the wound was closing, some hemorrhage recurred, while the patient was straining to evacuate the bowels.

The house surgeon, upon this, plugged the wound as had been done before; but blood then flowed by the urethra. The plug was therefore removed. The hemorrhage, after the loss of twelve ounces of blood, was finally arrested by the finger applied in the wound for an hour.

In Hearne's case, about eight ounces of blood were lost in the operation; the bleeding then stopped, as usual. Two hours afterwards, the bleeding recommenced, but was arrested by the dresser keeping his finger in the wound, upon the surface which bled. Mr. Mayo saw the patient an hour afterwards, when, the pressure being taken off, the hemorrhage did not recur then or subsequently. Mr. Mayo recommends, if hemorrhage recurs, pressure to be made with the fingers, as more effectual and less painful than the operation of plugging the wound.

Upon the subject of lithotrity, Mr. Mayo observed, that he was as yet far from being able to speak with confidence. The instruments which he uses are those invented by Civiale, and made by Charrière, with an additional and better perforator made by Everill and Mason; and a brise-pierre, made by Ferguson, upon the construction of that invented by M. Heurteloup. In the use of these instruments, Mr. Mayo deprecates the employment of a vice to fix them while in the bladder; the hand of an assistant being infinitely safer and more manageable than a vice, and, at the same time, sufficiently steady for the purpose. These instruments are used with great facility, and, in most instances in which Mr. Mayo has tried them, the stone has been seized in from fifteen seconds to a minute.

It does not appear that lithotrity is *materially* less painful than lithotomy. In lithotomy, the pain which the patient suffers, is caused less by the incisions that are made, than by the introduction of the forceps, the search after the stone, and its extraction: the suffering is from the contact, more or less violent, of the forceps with the bladder. That, which is the severest part of the cutting operation, is therefore in a degree common to lithotrity with it. Wareham, before he was cut by Mr. Wardrop, had had lithotrity ineffectually attempted by a French surgeon. He told Mr. Mayo that it was worse than the cutting afterwards; but he wished to have it tried again, as he thought it might be safer. Accordingly Mr. Mayo introduced Civiale's instrument; but the bladder was so exquisitely sensible, that he quickly withdrew it, and strongly advised his patient to be cut; which was done as above mentioned.

In Hearne's case, Mr. Mayo had several times used the lithotritic instruments, and much detritus had come away in the urine; and Mr. Mayo was anxious, knowing how small the portion which remained, to complete the cure of the patient in the way in which he had begun it; but the patient preferred being cut at once, and afterwards declared the pain of lithotomy to be less than that of the previous operations.

The objections to lithotrity are its painfulness, the probability that the operation will have to be repeated more than once, the uncertainty whether all the fragments are got rid of, and whether, after all, lithotomy may not become requisite. The advantages of lithotrity over lithotomy, are its *safety;* no danger, in this case, of hemorrhage, of infiltration of urine, and sloughing of the cellular membrane of the pelvis; or of the common risks incurred in every cutting operation, as, for instance, of death from erysipelas, or from the constitution sinking through unsuspected organic disease, and the like causes. Then, that the operation may be said to proceed at the pleasure of the patient, who may stop it at any instant, if the suffering become intense; that it does not render the other, if necessary afterwards, more severe; while it affords, if the stone be not large, a strong probability of its speedy destruction; holding out a chance of benefit, of which most of those who suffer from this malady, were they acquainted with it, would probably avail themselves.

